# Cystoid Macular Edema Secondary to Hyperviscosity Syndrome in Waldenström Macroglobulinemia: A Case Report of Multimodal Treatment Response and the Adjunctive Role of Acetazolamide

**DOI:** 10.1002/kjm2.70066

**Published:** 2025-06-20

**Authors:** Yu‐Mei Yang, Chuan‐Cheng Wang, San‐Ni Chen, Jian‐Sheng Wu

**Affiliations:** ^1^ Department of Medical Education Changhua Christian Hospital Changhua Taiwan; ^2^ Department of Internal Medicine Changhua Christian Hospital Changhua Taiwan; ^3^ Department of Ophthalmology China Medical University Hospital Taichung Taiwan; ^4^ Department of Ophthalmology Changhua Christian Hospital Changhua Taiwan; ^5^ Department of Post‐Baccalaureate Medicine National Chung Hsing University Taichung Taiwan

Waldenström macroglobulinemia (WM) is a rare lymphoplasmacytic lymphoma characterized by bone marrow infiltration and immunoglobulin M (IgM) monoclonal gammopathy [1]. We present a case of WM with ocular manifestations of hyperviscosity syndrome (HVS).

A 67‐year‐old man presented with bilateral blurred vision for 2 months. He was a hepatitis B carrier and had cataract in both eyes (OU). His best‐corrected visual acuity (BCVA) was 6/30 in the right eye (OD) and 6/120 in the left eye (OS). Fundus examination revealed retinal hemorrhages OU (Figure 1A,B). Fluorescein angiography (FAG) demonstrated delayed venous filling, mild leakage, mild optic disc congestion, and peripheral microaneurysms OU (Figure 1C,D). Optical coherence tomography (OCT) showed cystoid macular edema (CME) with serous retinal detachment OU (Figure 1E,F). Serum viscosity was 8.3 cP (cp), and IgM was 8860 mg/dL. Bone marrow biopsy showed lymphoplasmacytic cell infiltration with kappa light chain restriction. The diagnosis of WM with HVS and CME was established.

**FIGURE 1:**
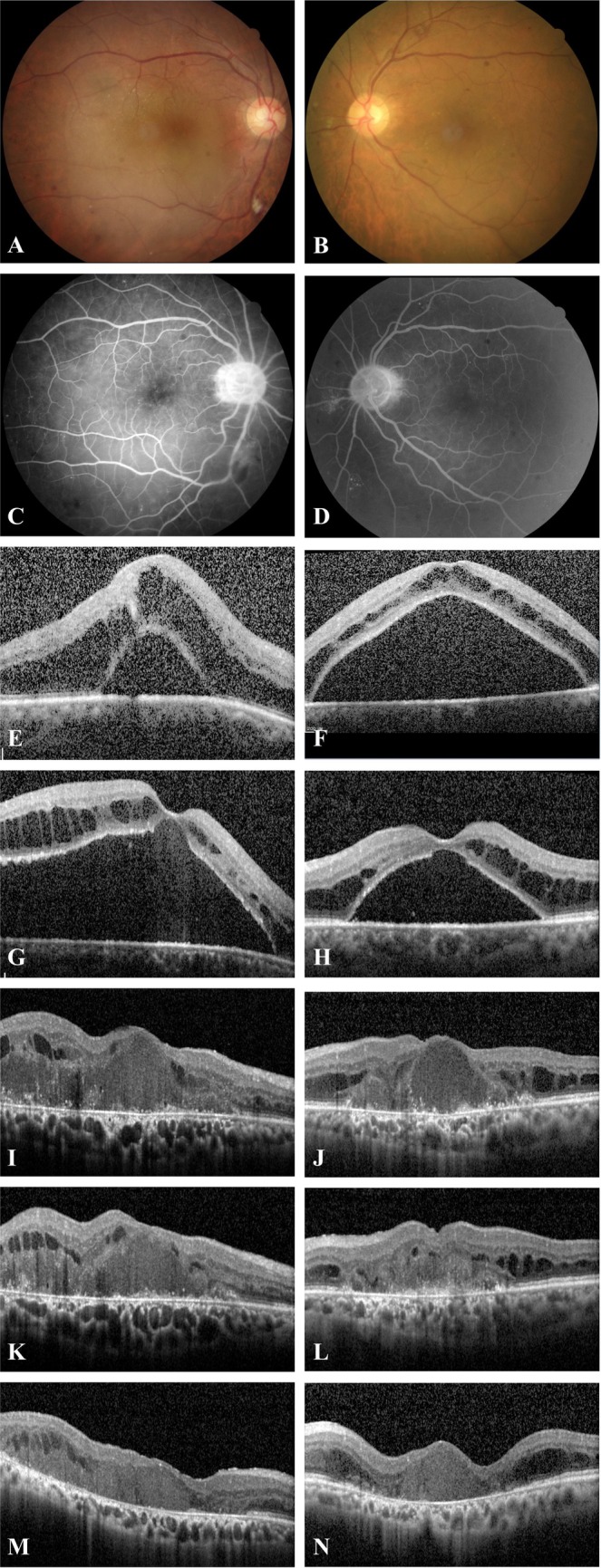
(A, B) Initial fundus examination revealed diffuse retinal hemorrhages OU. (C, D) Initial fluorescein angiography demonstrated delayed venous filling, mild leakage, mild optic disc congestion, and peripheral microaneurysms OU. (E, F) Initial OCT showed cystoid macular edema (CME) with serous retinal detachment OU. (G, H) After cyclophosphamide with steroids, intravitreal anti‐VEGF agents, and subconjunctival triamcinolone, OCT showed progressive subretinal fluid. (I, J) After bendamustine with rituximab and intravitreal triamcinolone, OCT showed improved subretinal fluid. (K, L) After plasmapheresis, OCT showed no significant changes. (M, N) After oral acetazolamide for 1 year, OCT showed resolution of edema.

Initial treatment included cyclophosphamide with steroids (prednisolone or dexamethasone) for systemic control, intravitreal anti‐VEGF agents (bevacizumab and ranibizumab), and subconjunctival triamcinolone for macular edema. This resulted in a minor response (IgM 5220 mg/dL, 41% reduction) but progressive subretinal fluid in OCT (Figure 1G,H). Six cycles of bendamustine with rituximab led to a partial response (IgM 1810 mg/dL, 79% reduction) with improved subretinal and intraretinal fluid and BCVA of 6/20 OU. Intravitreal triamcinolone was administered once, limited by elevated intraocular pressure.

Persistent macular edema prompted plasmapheresis. OCT and serum IgM showed no significant changes after plasmapheresis (Figure 1I,J: before plasmapheresis; Figure 1K,L: after plasmapheresis). Given the limited response, oral acetazolamide was added, supported by evidence from other retinal diseases [2, 3]. Resolution of edema (Figure 1M,N) and BCVA of 6/20 OD and 6/60 OS were noted after one year of acetazolamide at the final visit.

HVS typically occurs at serum viscosity > 4 cp (normal range: 1.2–1.8 cp) [1]. Systemic management includes plasmapheresis for rapid viscosity reduction and chemotherapy for hematologic control [1]. Local therapy like anti‐VEGF and corticosteroid injections partially mitigates vascular leakage, reducing intraretinal fluid [4]. However, subretinal fluid caused by IgM‐induced osmotic effects or direct accumulation is less responsive to VEGF‐targeted therapies [4]. Persistent subretinal fluid and chronic retinal damage to the ellipsoid zone and RPE degeneration limit full visual recovery [5].

Acetazolamide reduces aqueous humor production and intraocular pressure, potentially alleviating macular edema. Jia Liang et al. demonstrated superior anatomical and visual outcomes with oral carbonic anhydrase inhibitors (CAIs) compared to anti‐VEGF agents in patients with retinitis pigmentosa‐associated CME at 6 months [2]. Similarly, Yeo et al. found that CME involving the outer nuclear layer or central fovea was more likely to respond to CAIs [3]. These findings support the observed response in our case and the potential role of acetazolamide in managing refractory macular edema.

This case highlights the challenges of managing CME secondary to HVS in WM. Further resolution of edema was noted after adjunctive acetazolamide. Early intervention and consideration of adjunctive therapies may enhance outcomes. Further studies are warranted to validate the role of acetazolamide in this context.

## Conflicts of Interest

The authors declare no conflicts of interest.

## Data Availability

Data sharing not applicable to this article as no datasets were generated or analysed during the current study.
